# Krill oil protects dopaminergic neurons from age-related degeneration through temporal transcriptome rewiring and suppression of several hallmarks of aging

**DOI:** 10.18632/aging.204375

**Published:** 2022-11-09

**Authors:** Tanima SenGupta, Yohan Lefol, Lisa Lirussi, Veronica Suaste, Torben Luders, Swapnil Gupta, Yahyah Aman, Kulbhushan Sharma, Evandro Fei Fang, Hilde Nilsen

**Affiliations:** 1 Institute of Clinical Medicine, Department of Clinical Molecular Biology, University of Oslo, Oslo N-0318, Norway; 2 Section of Clinical Molecular Biology, Akershus University Hospital, Nordbyhagen N-1474, Norway; 3 Department of Microbiology, Oslo University Hospital, Oslo N-0424, Norway; 4 Department of Biosciences, University of Oslo, Oslo N-0318, Norway

**Keywords:** krill oil, aging, healthspan, mitochondrial health, senescence

## Abstract

There is accumulating evidence that interfering with the basic aging mechanisms can enhance healthy longevity. The interventional/therapeutic strategies targeting multiple aging hallmarks could be more effective than targeting one hallmark. While health-promoting qualities of marine oils have been extensively studied, the underlying molecular mechanisms are not fully understood. Lipid extracts from Antarctic krill are rich in long-chain omega-3 fatty acids choline, and astaxanthin. Here, we used *C. elegans* and human cells to investigate whether krill oil promotes healthy aging. In a *C. elegans* model of Parkinson´s disease, we show that krill oil protects dopaminergic neurons from aging-related degeneration, decreases alpha-synuclein aggregation, and improves dopamine-dependent behavior and cognition. Krill oil rewires distinct gene expression programs that contribute to attenuating several aging hallmarks, including oxidative stress, proteotoxic stress, senescence, genomic instability, and mitochondrial dysfunction. Mechanistically, krill oil increases neuronal resilience through temporal transcriptome rewiring to promote anti-oxidative stress and anti-inflammation via healthspan regulating transcription factors such as SNK-1. Moreover, krill oil promotes dopaminergic neuron survival through regulation of synaptic transmission and neuronal functions via PBO-2 and RIM-1. Collectively, krill oil rewires global gene expression programs and promotes healthy aging via abrogating multiple aging hallmarks, suggesting directions for further pre-clinical and clinical explorations.

## INTRODUCTION

Aging is a biological process characterized by progressive loss of viability and increasing frailty. As aging is the primary risk factor for many of the major diseases, it is generally accepted that slowing aging may reduce the incidence of age-related diseases [1]. There is mounting evidence that interfering with the conserved processes that induce aging can extend healthy lifespan [2]. However, many cellular processes contribute to aging. These processes are often referred to as “hallmarks of aging” and include genomic instability, epigenetic alterations, mitochondrial dysfunction, loss of proteostasis, senescence, telomere shortening, altered metabolism and cell-cell communication, as well as stem-cell exhaustion [3–5]. Anti-aging supplements should ideally attenuate most of these hallmarks to be effective.

Marine oils have been extensively researched for health promoting properties. However, the underlying molecular mechanisms underlying the health-promoting qualities of krill oil are not fully understood and a study that has evaluated the general potential of krill oil as a nutraceutical targeting the aging process is lacking. Extracts from the Antarctic krill species, *Euphausia superba*, have a high content of long-chain omega-3 fatty acids (including eicosapentaenoic acid (EPA) and docosahexaenoic acid (DHA)). In krill oil, EPA/DHA are mainly bound to phospholipids in contrast to fish oil, where they are only bound to triacylglycerol. These fatty acids are important in the brain with diverse roles ranging from maintenance of brain structure and function and serve as essential building blocks of healthy cell membranes. Phospholipids improve tissue uptake and facilitate efficient brain delivery [6]. The potent antioxidant and anti-inflammatory compound astaxanthin is a natural component of krill oil [7]. In a MPTP-induced Parkinson Disease (PD) mouse model, astaxanthin protected dopaminergic neurons in the nigrostriatal circuit in young mice, but not in older animals [8]. Krill oil is also a dietary source of the essential nutrient choline [7] which is a component of the phospholipid phosphatidylcholine (PC) and a precursor of the neurotransmitter acetylcholine [9]. Thus, due to the combination of several compounds with neuroprotective properties (choline, EPA/DHA and astaxanthin) [10, 11], exquisite bioavailability, krill oil may be a nutraceutical of choice to boost brain health [9]. In support of this, short-term supplementation experiments have shown that DHA/EPA has anti-inflammatory and anti-oxidative properties with the potential to improve motor function and some cognitive properties [12].

The nematode *Caenorhabditis elegans (C. elegans)* is regarded as a good model to study aging as most pathways affecting the aging processes are conserved between the nematode and humans [13]. *C. elegans* is a good model system to study the mechanisms of how the aging process leads to the development of age-related diseases. A specific formulation of krill oil was previously found to increase the lifespan in *C. elegans*, with the median lifespan increasing by about four days [14]. In this study, we used *C. elegans* to explore whether krill oil may also promote healthy aging. To evaluate the potential to attenuate age-related diseases we used PD as a model, because aging is the major risk factor for this neurodegenerative disease (NDD). We took advantage of transgenic strains expressing human α-synuclein (α-SYN) tagged with green fluorescent protein (GFP) in dopaminergic neurons and muscle [15–18]. These transgenic strains, facilitate visualization of age-related degeneration of dopaminergic neurons and aggregation of α-SYN - two cardinal pathologic features of PD. We found that krill oil counteracts the primary drivers of aging, such as mitochondrial dysfunction, senescence, and genomic instability in *C. elegans* and we validated the main findings in human fibroblasts.

## MATERIALS AND METHODS

### *C. elegans* strains and culture conditions

Animals were cultured on nematode growth medium (NGM) plates with the *Escherichia coli* strain OP50 at 20°C using standard procedure [19]. The adult animals were bleached to obtain synchronized populations. For all aging associated studies, L4 hermaphrodites were grown in NGM plates containing 0.5 μl/ml krill oil (provided by Aker BioMarine) from day 1 to day 6. The aging population of worms were maintained in NGM plates without FdUrd. Instead, animals were washed with M9 buffer and filtered through Nylon Net Filter (Millipore) everyday post adult day 2 stage, till the desired age. The adult day 1 was defined as 24 hours post L4 stage. The following nematode strains were used in this study: N2, wild type; BY273 *Is*[*p_dat-1_*GFP; p_*dat-1*_ α-syn] to monitor dopaminergic neurons. HLN107 strain was built by crossing TU3401: *sid-1 (pk3321) V; uls69 V* with BY273 to perform RNAi in dopaminergic neurons. *P_unc-54_ α-syn*::GFP was used to investigate α-synuclein aggregation. To study Alzheimer disease (AD) using *C.elegans* strain BR5270 [P_rab-3_::F3(delta) K280] and strain CK12 [aex-3::tau4R1N(P301L) + myo-2p::gfp] were used for Tau model; strain CL2355[P_snb-1_::Aβ_1–42_] was used for Aβ model; strain UM0001[P_snb-1_::Aβ_1–42_;P_rab-3_::F3(delta) K280] was used for Tau; Aβ model. In the following, the N2 and BY273 strains will be referred as wild type (WT) and PD animals respectively. The strains BR5270, CK12, CL2355 and UM0001 will be referred to as AD animals.

### Cell lines

The human fibroblastoid cell line BJ was grown in Dulbecco’s Modified Eagle Medium, GlutaMAX (DMEM, Life Technologies) supplemented with 10% (vol/vol) fetal bovine serum (FBS, Lonza) and 1× Penicillin-Streptomycin (Life Technologies). Cells were grown at 37°C with 5% CO2.

### RNA isolation and qPCR

Total RNA was isolated with RNeasy mini kit (Qiagen) following the manufacturer’s instructions. Reverse transcription was performed using High-Capacity cDNA Reverse Transcription kit (Applied Biosystems). Quantitative PCR was carried out on a QuantStudio 7 Flex detection system (Applied Biosystems) with the Power SYBR green PCR master mix (Applied Biosystems). Each sample was analysed in triplicate. Primer sequences are provided in Supplementary Table 1.

### Chemicals and antibodies

The senescence marker X-gal were purchased from Sigma (B4252-250MG). Serotonin hydrochloride was from Sigma. The following commercially available antibody was used: monoclonal 8-oxo-dG (4354-MC-050 Trevigen). Secondary antibodies were Alexa Fluor 555-conjugated anti-rabbit and anti-mouse (Invitrogen). For RNA isolation, Direct-zol RNA Miniprep Kits (#R2050) was used from Zymo-research. Senescence marker for fibroblasts, SPiDER-β-gal, was purchased from Dojindo (SG02-10). TMRE, (Mitochondrial membrane potential assay kit) was purchased from Abcam (ab113852). Cell nucleus staining dye Hoechst was purchased from Thermo Scientific (Hoechst 33342).

### Degeneration of dopaminergic neurons

The degeneration of dopamine neurons in BY273 Is[p_dat-1_GFP; p_dat-1_ α-syn] was monitored by following GFP expression under the dopamine transporter (DAT-1) promoter [20, 21]. The L4 larval stage of BY273 animals were exposed to vehicle and krill oil (0.5 μl/ml media). The *in vivo* imaging of dopaminergic neurons was performed on day 1, day 3 and day 6 old animals. At this stage, 15–20 worms were immobilized on an agar padded glass slide with 2 mM levamisole and glass cover slip. The dopaminergic (DA) neurons were imaged under a Zeiss LSM780 confocal microscope with ×20 objective.

### Immunohistochemistry

For immunostaining, adult worms were washed twice with milliQ water. Washed worms were placed on poly-L-lysin-coated slides (Thermo Scientific), and freeze cracked using coverslips on a dry ice block. The primary 8-oxo-dG antibody was used at 1:200 dilution. The secondary antibody, Alexa Fluor 555-conjugated anti-mouse was used at 1:1500 dilution. Prolong gold with DAPI was used for mounting (Invitrogen, P36931). The slides were imaged under Zeiss LSM780 confocal microscope with ×63 plan-Apochromat 1.4 NA objective.

### Basal slowing response

The basal slowing response was performed as described [20]. Well-fed synchronized old day 6 worms were tested. The NGM plates with and without OP50 were used to count body bends per 20 seconds. The locomotion rate was counted after 5 minutes of transfer, to avoid overstimulation. The assay was performed blindfolded, with three independent replicates.

### Pharyngeal pumping

We used a microfluidics based ScreenChip sytem (*In vivo* Biosystem) to detect pharyngeal pumping rate in each individual worm [20]. For this assay worms were age-synchronized. The worms were first washed twice with M9 buffer, before incubating them with 10 mM serotonin (prepared with M9 buffer) at room temperature for 30 minutes. Simultaneously, the Screenchip fluidics system was prepared by following the user guide. The worms were loaded in 1.5 ml Eppendorf tubes with 1 ml M9 buffer and vacuum sucked into the screen-chip using a vacuum pump. Pumping frequency was measured by EPG recording using screen-chip 40 for old day 3 worms. Each recording was 1 to 2 minutes long, with 15–20 worms per genotype.

### Chemotaxis associated learning

The short term associated memory training (STAM) assay was performed as described in [20, 22]. The assay is based on the ability of *C. elegans* to learn and remember a positive association between food and the weak chemoattractant butanone. Briefly, age-synchronized worms maintained at 20°C were divided into naïve and trained groups. Each group had 200 to 300 worms per condition. The trained group was washed three times with M9 and starved for one hour in M9 before training. After two hours of conditioning, the worms were trained to positively associate food and butanone, they were kept at hold for 120 minutes in seeded NGM plates. STAM was measured after two hours intervals of (spaced) training. Worms were tested for chemotaxis toward 10% butanone before (naïve) or after two hours conditioning (trained). The chemotaxis index was calculated as follows: (CI) = (n _butanone_ − n _ethanol_)/(Total − n _origin_). Learning Index was calculated by subtracting the naïve CI from the post-conditioning training CI (CI_butanone_-CI_naive_). The memory assay in AD animals was performed as described elsewhere [23].

### Oxygen consumption rate

The oxygen consumption rate was measured using DW1/AD clark-type polarographic oxygen sensor (Hansatech Instruments, model: Oxygraph Plus System). The protocol was exactly implemented as described in [24] without modification.

### Senescence

The senescence assay was performed as described in [25] with minor modifications. Briefly, the animals were washed with PBS and fixed with 0.2% glutaraldehyde + 2% PFA in 1xPBS on dry ice for 5 minutes. Next, the animals were thawed at room temperature for 10 minutes and stained with freshly prepared X-gal staining solution containing 1 mg/ml X-gal (stock 20 mg/ml in DMSO), 40 mM citric acid/sodium phosphate pH 6.5, 5 mM potassium ferricyanide, 150 mM NaCl and 2 mM MgCl2 and incubated overnight at 37°C covered in dark. The stained animals were washed with 1xPBS and mounted on glass slides with coverslips. The animals were imaged under a bright field microscope with ×20 objective.

Senescence assay in BJ fibroblasts was done using SPiDER-β-Gal marker kit. 50,000 (late passaged) fibroblasts cells were seeded on 35 mm imaging dish (IBIDI) with polymer coverslip bottom and cells were allowed to grow overnight at 37°C in 5% CO2 incubator. 24 hours post seeding, cells were treated with 100 μg/ml krill oil and allowed to grow for 6 days at 37°C in 5% CO2 incubator. Senescence assay on live cells was performed on day 7 according to the manufacturer's protocol. Briefly, cells were washed once with PBS and then treated with 1 ml of Bafilomycin working solution and incubated at 37°C for 1 hour in a 5% CO2 incubator. After 1 hour, 1 ml of SPiDER-β-Gal working solution containing 2 μg/ml Hoechst was added to the cells and they were incubated again for 30 minutes in a 5% CO2 incubator. After incubation, cells were washed with PBS and then imaged under Zeiss LSM780 confocal microscope at ×63 with appropriate filters.

### TMRE staining

A TMRE (Tetra methyl rhodamine, ethyl ester) Mitochondrial Membrane Potential assay kit was used to measure the mitochondrial membrane potential. In total, (late passaged) 50,000 BJ fibroblasts cells were plated onto 35 mm imaging dish (IBIDI) with polymer coverslip bottom. 24 hours post seeding cells were treated with 100 μg/ml krill oil and the cells were allowed to grow for another 5 days at 37°C in 5% CO2 incubator. On day 6, TMRE assay was performed according to the manufacturer's protocol. Briefly, cells were stained at a final concentration of 200 nM TMRE and incubated in dark for 30 min at 37°C. After incubation, cells were washed again with PBS and live cell imaging was done on Zeiss LSM780 confocal microscope, at ×63 magnification using appropriate filters.

### Mitochondria copy number and gene expression analysis

Mitochondrial DNA (mtDNA) copy number was quantified using droplet digital PCR (ddPCR). Briefly, the age-synchronized wild type and PD animals were individually picked in lysis buffer (TE low containing 1 mg/ml proteinase K and 0.01 mg/ml RNase). The lysis was performed for 1 hr at 65°C followed by 95°C for 15 minutes. From here this method was performed exactly as described in [20].

For mitochondrial gene expression analysis, transcriptional activation of ctc-1 and nduo-2 was measured in age-synchronized wild type and PD animals. The single worms were collected in 1 ml PBS and immediately snap frozen in dry ice. The protocol was exactly implemented as described in [20] without modification.

### RNA isolation for RNAseq

Age-synchronized wild type and PD animals were grown on regular medium of medium supplemented with krill oil. These animals were harvested as day 1, day 3, day 6 adults and RNA isolation was performed the same day. Briefly, the animals were washed ×2 with sterilized milliQ water, and collected in 1.5 ml Eppendorf. The worm pellets were dispensed in 600 μl of Trizol (Sigma) and transferred to tube with sterile beads to homogenize the animals. From here RNA isolation for tissue was followed as described in the Direct-zol RNA Miniprep Kits protocol section. The isolated RNA was dissolved in 10 μl nuclease free water and the quality was analyzed in bioanalyser.

### RNA sequencing

Sequencing libraries were prepared from 200 ng total RNA using Nugen Universal Plus Total RNA-Seq library preparation kit (Tecan) with custom AnyDeplete design for *C. elegans*. Final libraries were pooled (10 samples/run) and paired-end sequencing (2 × 71 bp) performed using NextSeq High Output kits on the NextSeq 550 sequencer. The raw sequencing data were demultiplexed using BCL Convert (Illumina). Sequencing data preprocess starts right after being demultiplexed. Sequences were aligned to *C.elegans* WBcel235 DNA reference (release-96) [26] using annotations, general transfer format (.gtf) file from the same release, and with STAR aligner (version 2.7.10a) [27]. First, an index was created using parameters --runMode genomeGenerate, --runThreadN 8 --genomeSAindexNbases 12, --sjdOverhang 69, and for any other parameter the default, in the specified version, was used. Second, reads were aligned using parameters --runThreadN 8, --outSAMtype BAM SortedByCoordinate --sjdbOverhang 69 and the ones suggested by STAR manual in ENCODE options section --outFilterType BySJout, --outFilterMultimapNmax 20, --alignSJoverhangMin 8, --alignSJDBoverhangMin 1, --outFilterMismatchNmax 999, --outFilterMismatchNoverReadLmax 0.04, --alignIntronMin 20 --alignIntronMax 1000000 --alignMatesGapMax 1000000. From the BAM file obtained we created an index using samtools version 1.15.1 [28] so we could remove UMI-duplicates using umi-tools version 1.1.2 [29], with parameters --log2stderr, --paired, --umi-separator=”:”. We then sorted the deduplicated BAM file by name and created fastq files with the same parameters -@ 4, -n.

### RNA seq data analyses

Differential gene expression was performed using the DESeq2 R package [30], version 1.32.0. Count files were split into their respective groups and attributed defining conditions, such as ‘control’ and ‘experiment’. The counts for the respective conditions are merged into one dataframe; from this dataframe genes which have no counts for every sample are removed. A sample defining file containing the sample names along with their associated condition is also created, this file contains the relevant information for the samples within the merged count file. The values are then normalized using the DESeq2 normalization method. The standard DESeq2 pipeline was used to obtain the differential gene expression results. It starts by creating the DESeqDataSet-class by calling the DESeqDataSetFromMatrix function, using the merged count file, and the sample defining file are used as inputs, while the condition is used as the design. The design always uses the ‘experiment’ and compares it with the ‘control’. Using the estimateSizeFactors function, the median ratio method is utilized to estimate the size factors of the DESeqDataSet object. The DESeq2 function is called, which performs an estimate of dispersion followed by a negative binomial generalized linear model fitting and wal statistics in order to obtain the differential gene expression results. Selection of significant differentially expressed genes was made based on an adjusted p value (false discovery rate) below 0.05 and a log2-fold change value greater than 1 or smaller than -1.

The time series analysis was performed using a package developed in our lab (Lefol et al., manuscript in preparation). The package takes in raw RNAseq files and creates a matrix of raw counts, these counts are then normalized using the DESeq2 method [30]. It then performs differential gene expression analyses for both the conditional and temporal elements of the time series analysis, where conditional elements are the comparison of the experiment versus the control at every time point. Temporal elements are the comparison of subsequent time points, where the later time point is used as the experiment and the earlier time point is used as the control. The significant genes from all differential gene experiments are pooled together and clustered using the clusterGenomics (Identifying clusters in genomics data by recursive partitioning package, version 1.0.). A functional enrichment of each cluster is obtained using gprofiler [31].

The WGCNA package [32], version 1.70–3 was used to obtain a weighted correlation association between gene modules and behavioral parameters. WGCNA takes in RNAseq count data. A sample file was manually prepared for the WGCNA package, the sample file details which behavioral values are associated with the samples inputted. The pipeline first removes any samples with too many missing values in proportion with the amount of samples and values inputted. The samples are then clustered and their power is evaluated. Based on the indications of [32], the power value used is the first value above the 0.8 threshold which was a value of 4. The blockwise Modules function is used to calculate the gene modules and obtains the correlation values with the clinical parameters. Each gene module is run through gprofiler [31] in order to extract potential biological relevance of each gene module.

Biological age predictions were performed using the fastq files as raw data as input in the preprocessing specified in the Bit age pipeline [33]. First, the data were processed with fastp version 0.23.2 [34] with parameters -g -x -q 30 -e 30 -w 8. And second, reads were aligned with STAR aligner and parameters --quantMode GeneCounts --runThreadN 8, --outSAMtype BAM Unsorted --sjdbOverhang 69 and again the ones suggested by STAR manual in ENCODE options, as above. For predicting biological age we took raw counts from the STAR aligner output and computed count per million (CPM), as required by bit age method, with edgeR library version 3.38.1 [35]. We then computed predicted biological age using elastic net coefficients and code provided in the bit age material [33].

### Data availability

The data discussed in this publication have been deposited in NCBI’s Gene Expression Omnibus (Edgar et al., 2002) and are accessible through GEO Series accession number GSE207152 (https://www.ncbi.nlm.nih.gov/geo/query/acc.cgi?acc=GSE207152).

## RESULTS

### Krill oil protects dopaminergic neurons from age-related degeneration and improves dopamine-dependent behavior

**Figure 1:**
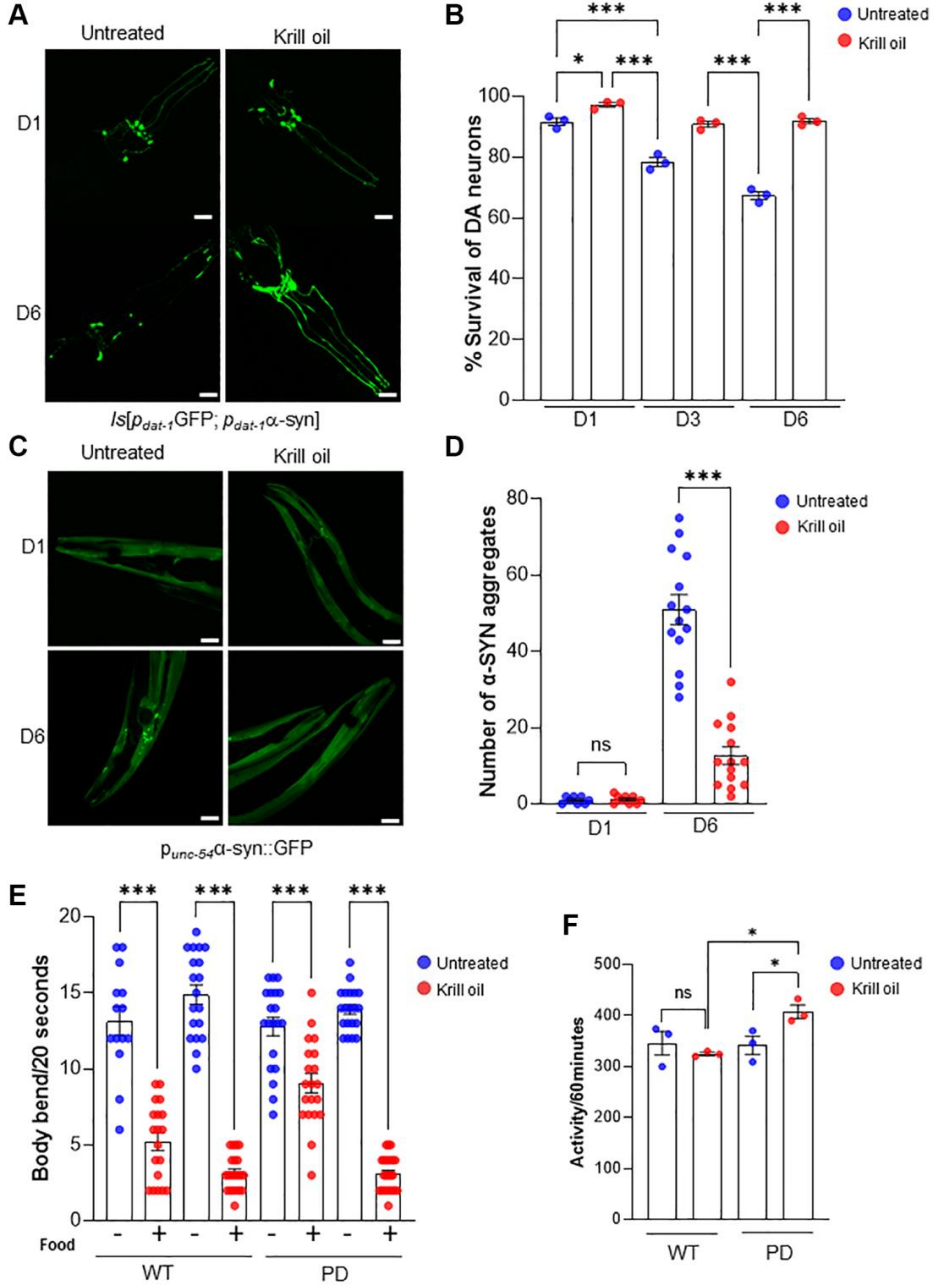
**Krill oil promotes healthy dopaminergic neuronal aging.** (**A**) Representative images of the head region of PD animals at day 1, day 3 and day 6 of adulthood, Scale bar, 20 μm. (**B**) Survival of anterior CEPs and ADEs DA neurons during aging in response to Krill oil (*n* = 35 nematodes per experiment; three independent experiments, s.e.m; ^***^p < 0.001; one-way ANOVA followed by Bonferroni’s multiple comparison test. (**C**) Representative images of α-SYN aggregation in body wall muscles in day 1 and day 6 animals, Scale bar, 20 μm. (**D**) The number of aggregates in day 1 and day 6 animals in response to krill oil (*n* = 15 nematodes, s.e.m; NS and ^***^p < 0.001; one-way ANOVA followed by Bonferroni’s multiple comparison test). (**E**) Column scatter plot representing basal slowing response of wild type and PD animals at adult day 6. Body bends per 20 second measured on NGM plates with and without bacteria (*n* = 30; Error bars, s.e.m; ^***^p < 0.001; one-way ANOVA followed by Bonferroni’s multiple comparison test. (**F**) Column scatter plot representing locomotion activity wild type and PD animals at adult day 6. The activity was scored for 60 minutes (*n* = 50 animals per experiment; Error bars, s.e.m; NS and ^*^p < 0.05; one-way ANOVA followed by Bonferroni’s multiple comparison test.

To determine whether growth in the presence of krill oil protected dopaminergic (DA) neurons from degenerating over time, we used the *C. elegans* PD model, a humanized strain, where overexpression of α-SYN under control of the *dat-1* promoter exacerbates age-dependent degeneration of DA neurons [15, 20]. As previously reported, we saw a substantial reduction of the number of DA neurons in adult day 6 old animals compared to the younger adults (day 1 and day 3) both when assessed by fluorescence intensity of the DA neuronal GFP (Figure 1A) and by scoring the number of DA neurons by differential interference contrast (DIC) microscopy (Figure 1B). Growth in media supplemented with krill oil resulted in a remarkable protection of DA neurons from age-related degeneration (Figure 1A and 1B). In comparison, aging reduced the fraction of DA neurons to less than 70% in day 6 adults compared to 90% in day 1 adults when grown on standard media. In contrast, there was no reduction of surviving DA neurons at day 3 to day 6 in krill oil-treated animals (Figure 1A and 1B). Moreover, there was a small increase in the fraction of surviving DA neurons in day 1 animals, suggesting a general protection against α-synuclein mediated proteotoxicity.

To test whether krill oil suppressed α-synuclein aggregate formation, a key pathogenic hallmark of Parkinson’s disease, we used a reporter strain expressing α-synuclein coupled with GFP in body wall muscles [20]. Day 1 young animals showed no evidence of α-synuclein aggregation, whereas the day 6 old animals had clear presence of α-synuclein aggregates, averaging about 50 aggregates per animal. Interestingly, krill oil treated animals showed a significant reduction of α-synuclein aggregation, with an average of about 17 aggregates (Figure 1C and 1D).

Dopamine-regulated behaviors are well characterized [36], and it has been demonstrated that DA neuron-dependent behavior, such as basal slowing response (BSR) and movement ability, deteriorates with age [20]. To determine whether improvement of DA neuron survival led to preservation of DA neuron function, we examined BSR in day 6 old wild type and PD animals. Intriguingly, krill oil-fed animals had significantly higher BSR than untreated animals (Figure 1E). Further supporting improved DA–dependent behavior performance, day 6 old animals showed an average activity score of 400/hour as compared to only 340/hour in untreated PD animals. Whereas the wild type animals showed no change in response to krill oil (Figure 1F). Thus, krill oil inhibits α-synuclein aggregate formation, and improves DA neuron survival and dopamine dependent behaviors in old animals.

### Krill oil reduces senescence in *C. elegans* and human fibroblasts

**Figure 2:**
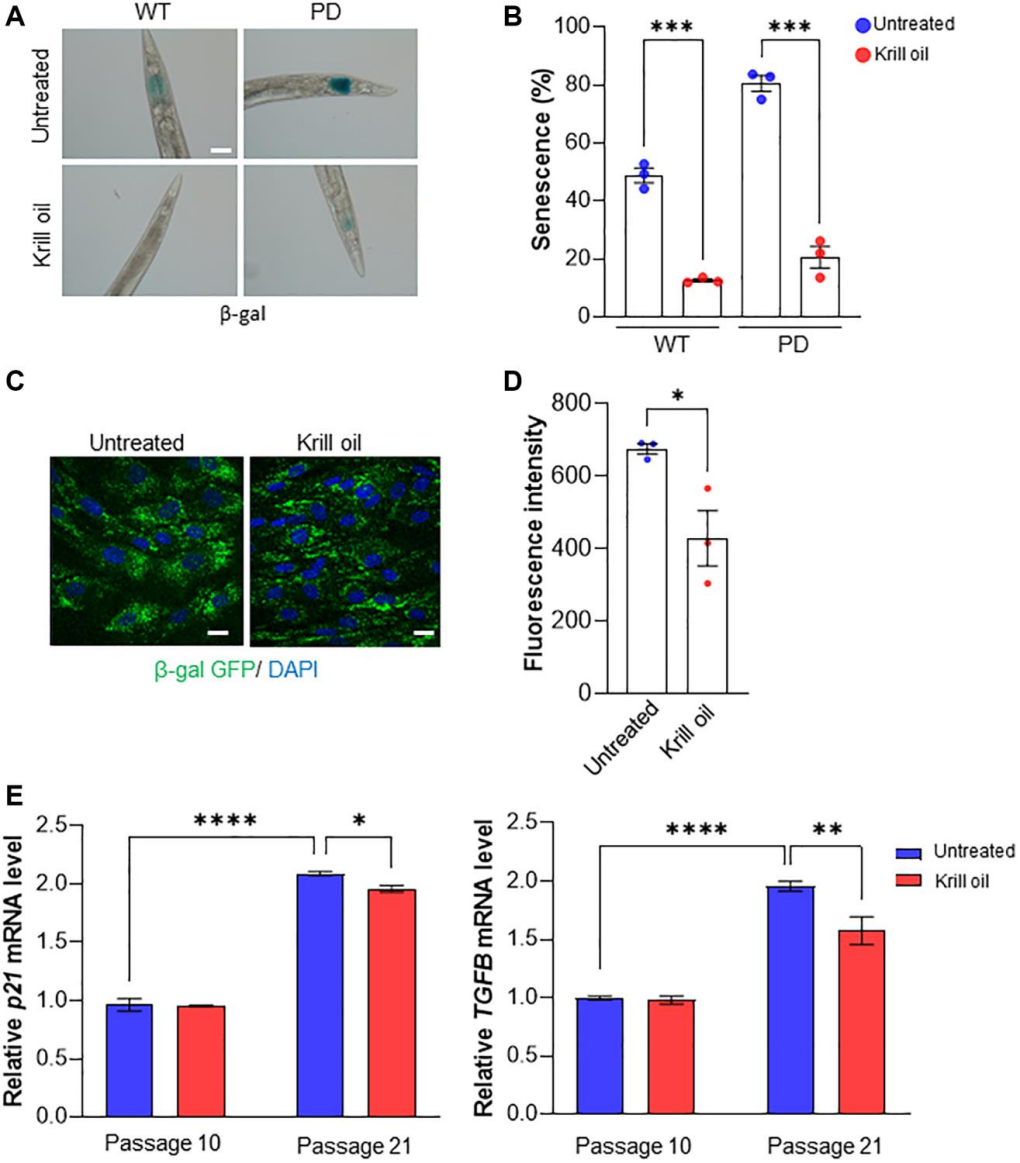
**Krill oil delays senescence.** (**A**, **B**) Representative images of β-gal staining in the head region in 9-day old adults, Scale bar, 20 μm. Column scatter plot representing the percentage of worms with positive senescence staining in three independent experiments (*n* = 50–100 individuals, column indicates mean, error bars, s.e.m, ^***^p < 0.001; one-way ANOVA followed by Bonferroni’s multiple comparison test). (**C**, **D**) Images and quantification represents senescence in late passage BJ cells using β-gal staining (three independent experiments, Scale bar, 20 μm, Error bars, s.e.m; ^*^p < 0.05; one-way ANOVA followed by Bonferroni’s multiple comparison test). (**E**) Relative *p21* and *TGFβ* mRNA levels in BJ cells at passage 10 and 21 treated, or not, with Krill oil (100 μg/ml, 6 days) as measured by qPCR. Data represent means ± s.d., *n* = 3. ^*^P ≤ 0.05, ^**^P ≤ 0.01, ^****^P ≤ 0.0001 (two-tailed Student’s *t*-test).

Senescence is a hallmark of aging and evidence suggests that senescence-like atrophy may be responsible for age-dependent loss of germline gonadal cells and self-destruction of intestinal biomass in *C. elegans* [37–39]. In 9-day old animals, 45% of wild type animals and 80% of PD animals exhibited positive β-gal staining. When these animals were fed krill oil, wild type and PD animals showed 35% and 60% reduction of positive β-gal staining, respectively (Figure 2A and 2B).

Since it is unclear whether *C. elegans* experiences a process similar to the senescence-associated secretory phenotype, which is an effector of senescence-driven aging in human tissue, we used human fibroblasts to validate whether krill oil counteracts senescence. When treated with krill oil, late passage BJ fibroblasts, which are widely accepted as a model to follow senescence, exhibited a 1.75-fold reduction in cells positive for β-gal, which is a marker of senescent cells (Figure 2C and 2D) as compared to the controls. The reduction in senescence in late passage BJ cells were accompanied by reduced expression of the senescence markers *p21* and *TGFβ* (Figure 2E).

### Krill oil improves mitochondrial health

**Figure 3:**
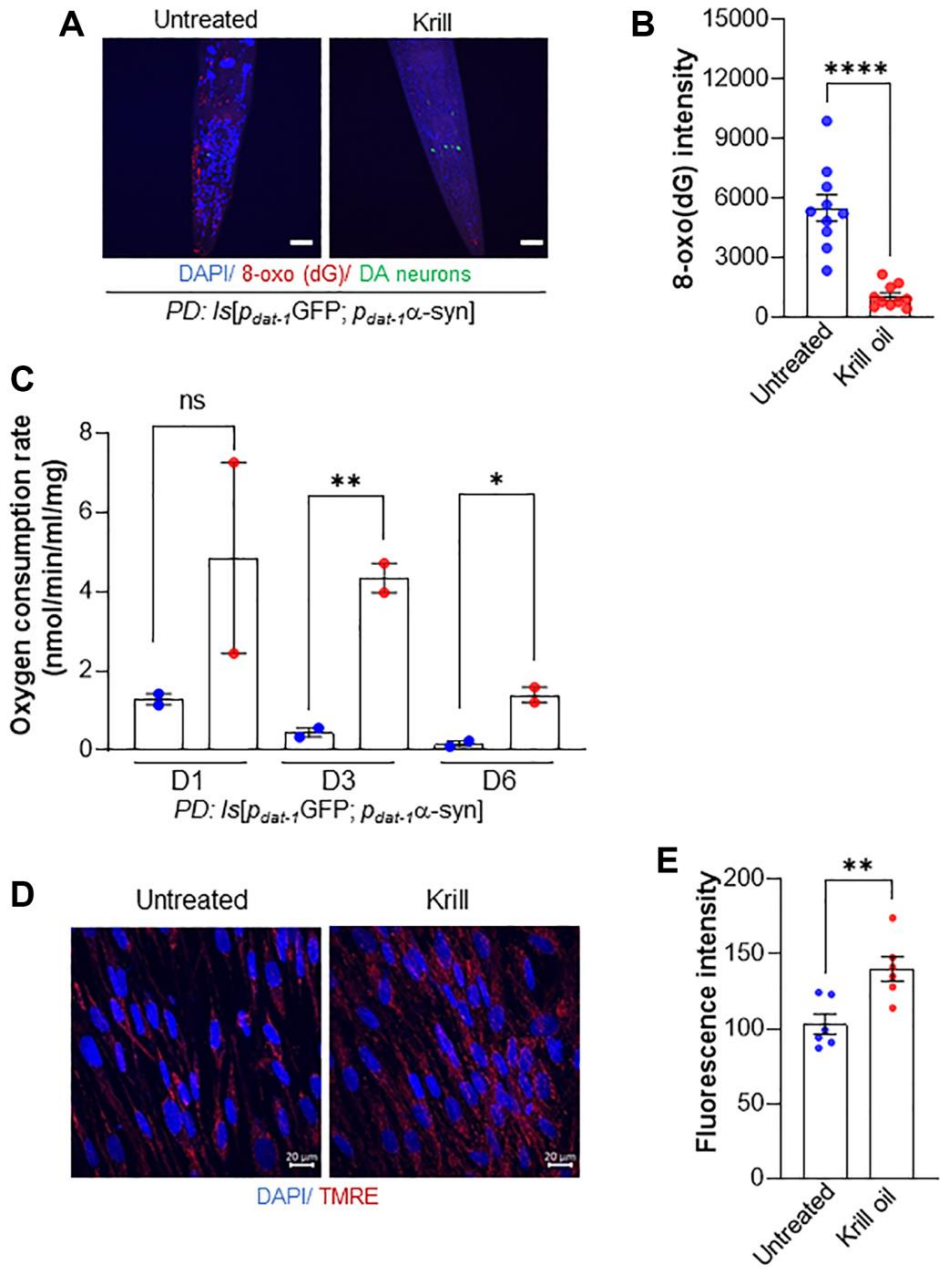
**Krill oil improves mitochondrial health.** (**A**, **B**) Representative image and quantification of 8-Oxo (dG) staining in old day 6 PD animals (*n* = 10 individuals, Error bars, s.e.m; ^****^p < 0.0001; one-way ANOVA followed by Bonferroni’s multiple comparison test). (**C**) Oxygen consumption rate in day 1, day 3 and day 6 PD animals (*n* = 50 individuals, two independent experiments, Error bars, s.e.m; NS and ^*^p < 0.05, ^**^p < 0.01; one-way ANOVA followed by Bonferroni’s multiple comparison test). (**D**, **E**) Image and quantification of mitochondrial membrane potential measured using TMRE staining in BJ fibroblast at passage 16 (p16) in the absence and presence of krill oil (*n* = 6 independent experiments, Scale bar, 20 μm, Error bars, s.e.m; ^**^p < 0.01; one-way ANOVA followed by Bonferroni’s multiple comparison test).

Oxidative stress is a fundamental feature of aging and a pathogenic factor in PD [3, 20]. Reactive oxygen species (ROS) generated due to metabolic activity by mitochondria are an important source of oxidative stress in brain cells. ROS cause oxidative DNA damage in both nuclear and mitochondrial DNA [40]. Oxidized guanine, 8-oxoG, is a biomarker of nucleic acid oxidation and oxidative stress. As previously demonstrated [20], old PD animals accumulate 8-oxoG in their body (Figure 3A and 3B). Because DHA/EPA and astaxanthin are components of krill oil with antioxidant properties, we were interested to see how 8-oxoG levels were affected. Compared to untreated animals, krill oil supplemented day 6 old PD animals showed a 6-fold decrease in 8-oxoG levels.

Mitochondrial dysfunction is a main source of oxidative stress, and can be followed by measuring the oxygen consumption rate (OCR) [24]. While the expected age-related reduction in OCR was seen in PD animals from day 1 to day 3, a drop in OCR was only apparent in day 6 old animals fed krill oil (Figure 3C). This was not attributable to a rise in mitochondrial copy number which remained relatively stable in both wild type and PD old day 6 animals fed on krill oil (Supplementary Figure 1).

To test whether krill oil also promoted mitochondrial health in human cells, we measured the mitochondrial membrane potential using tetramethylrhodamine ethyl ester (TMRE), a dye that is imported into mitochondria as a function of the mitochondrial membrane potential and, thus, stains healthy mitochondria. After only 5 days of treatment with krill oil, late passage human BJ fibroblasts showed a 1.2-fold increase in TMRE fluorescence intensity (Figure 3D, 3E).

### Krill oil induces temporal transcriptome rewiring

**Figure 4:**
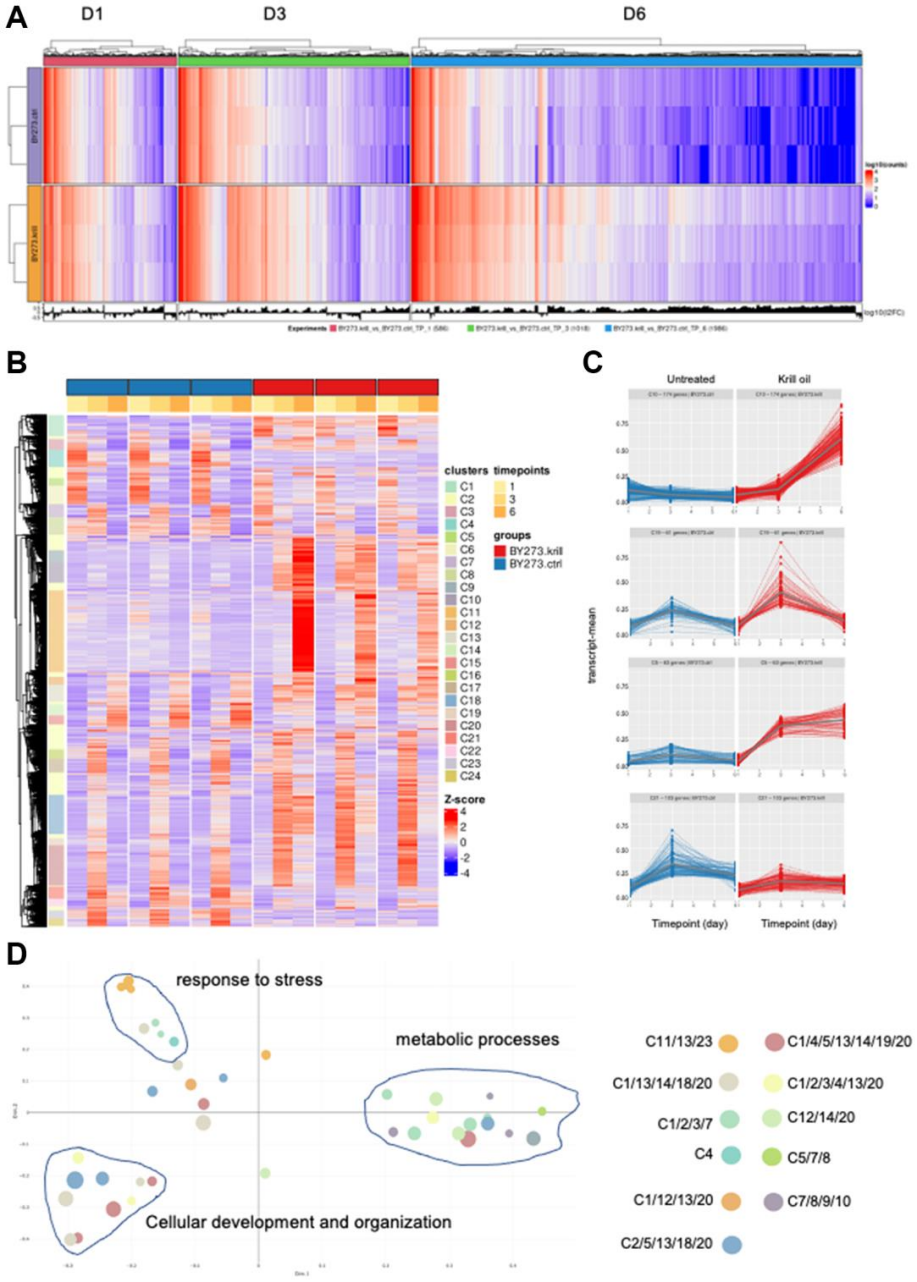
**Krill oil alters genes regulation.** (**A**) Differentially expressed genes (DEG) in PD animals in the presence and absence of krill oil treatment in day 1, day 3 and day 6 old animals. (**B**) Heat map of the time series analyses comprising all DEGs revealing 24 co-regulated clusters. (**C**) Examples of clusters (C10, C19, C5 and C21) with typical trajectories in PD animals. (**D**) Represents multi-dimensional scaling (MDS) plots showing all GOs and separating them based on clusters having similar functions.

In the above, we showed that krill oil has a widespread impact on many phenotypic hallmarks of aging. To get an unbiased view of the pathways activated by krill oil in a life-course perspective, we used RNA sequencing. Data was collected from day 1, 3, and 6 old wild type and PD animals. Principal component analysis (PCA) analyses showed good concordance between biological replicates and good separation of experimental groups with respect to age and treatment (Supplementary Figure 2A). Differential gene expression analyses showed that there was a successive increase in the number of differentially expressed genes (DEGs) in both strains (Figure 4A and Supplementary Figure 2B, 2C). In the PD strain, the number of upregulated DEGs increased from 586 DEGs at day 1 to 1018 in day 3 and 1986 in day 6.

To identify groups of genes that respond similarly to krill oil in a temporal manner, we performed gene cluster analysis comprising all DEGs with an absolute log2 fold change greater than one, as illustrated in a heatmap of all the genes submitted for PART clustering (Figure 4B). Several clusters with genes temporally regulated in response to krill oil were identified (Figure 4B), that could be broadly categorized into four main types of cluster trajectories (Figure 4C and Supplementary Figure 2D). Genes upregulated in day 6 old krill oil fed animals were found in clusters C7-C10. Genes upregulated in the animals at day 3 and day 6 were found in clusters C3-C6 and C17. Genes upregulated at day 3 only are exemplified in cluster C19, and finally, genes downregulated on day 3 and day 6 are represented by C21, C23-C24. Functional enrichment analyses performed on each cluster separately revealed similarities between clusters with similar trajectories (Supplementary Figure 3), where genes upregulated in old animals were dominated by genes involved in metabolic processes, e.g., protein metabolic processes. Clusters upregulated at day 3 were dominated by biological processes responding to stress, such as unfolded protein response and immunity. Clusters upregulated on day 3 only comprised several clusters with 1352 genes, but only C19 showed enriched biological processes. Interestingly, these were directly related to neuron function, such as cell projection assembly. Similarly, of genes downregulated exclusively at day 3, only cluster 23 showed enriched biological processes representing various defense responses.

To illustrate the relationship between the different clusters, we prepared Multi-Dimensional Scaling (MDS) plots that shows all the terms of the specified ontology (biological processes (BP)) and distinguishes the cluster(s) using colour and each terms relatedness using distance. Using a nearest ancestor clustering approach, where all terms found are brought up to their nearest common ancestor, the clusters fell nicely into three groups (Figure 4D). This approach clearly shows the relatedness between the GOs involving neurogenesis, neuron projection morphogenesis, and cell projection organization (representing C13, C14 and C20 illustrated by light grey balls), and developmental processes (illustrated with red, blue and yellow balls in the lower left quadrant). Response to stress and immunity are represented in the top left quadrant whereas cellular and macromolecular metabolic processes fall together (Figure 4D).

### Transcriptome rewiring promotes neuron survival

**Figure 5:**
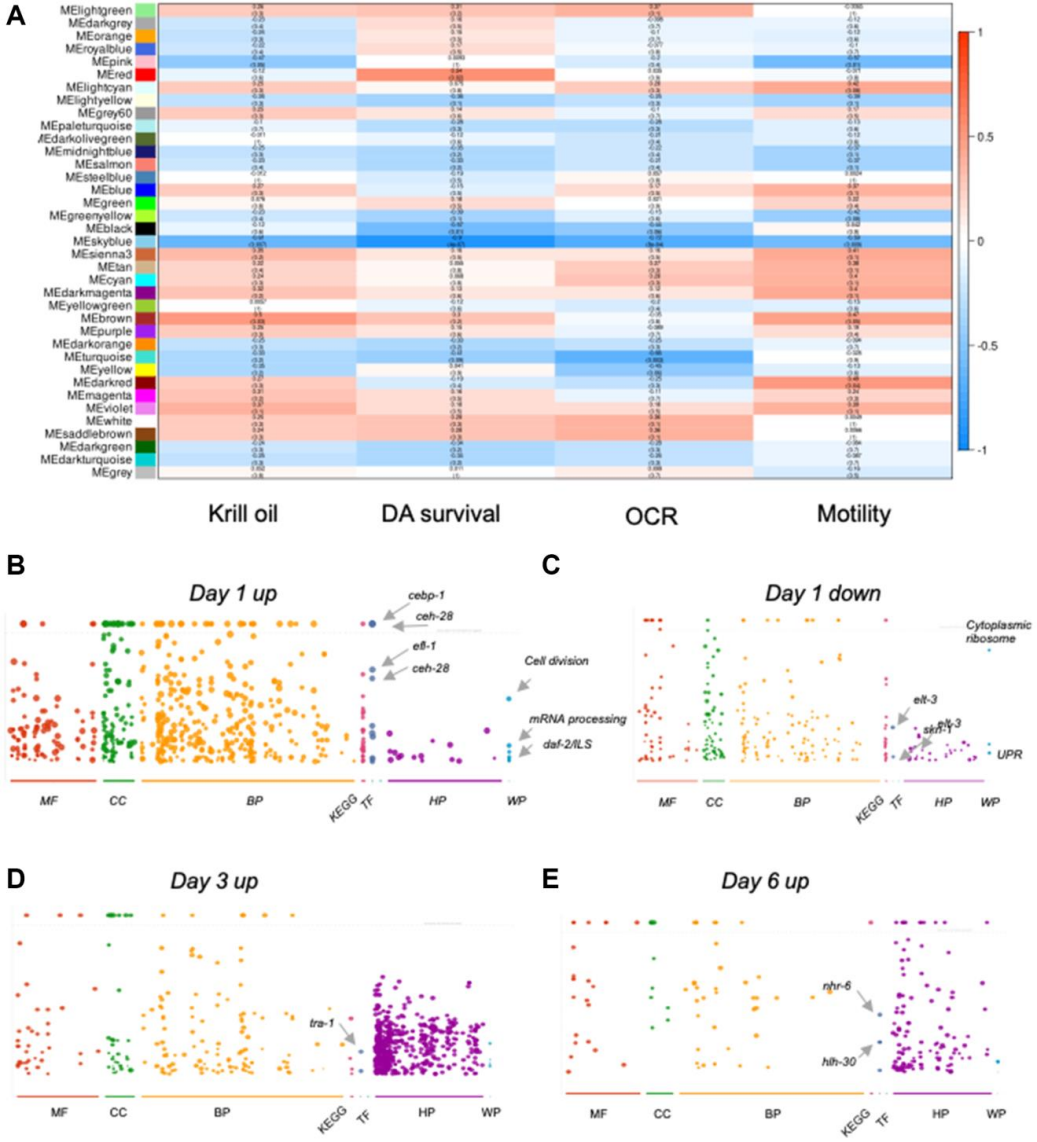
**Krill oil rewires gene expression to promote neuron survival.** (**A**) Heatmap from WGCNA analysis shows the correlation values between all of the found modules (gene groups) and defined parameters (krill oil treated, DA neuron survival, OCR and locomotion). The correlation ranges from +1 (completely positively correlated) to -1 (perfect negative correlation). (**B**–**E**) Gprofiler plots with functionally enriched transcription factors indicated; (**B**) Representation of the modules (turquoise, white and saddlebrown) upregulated in day 1 PD animals. (**C**) Representation of the modules (red, pink and dark grey) downregulated in day 1 PD animals. (**D**) Representation of the modules (blue, tan, cyan, light cyan, violet, dark magenta, sienna) upregulated in day 3 PD animals. (**E**) Representation of the modules (magenta, purple, dark olive green, and yellow green) upregulated in day 6 PD animals.

To further narrow down which components of the global transcription reprogramming correlate with changes in phenotypic end-points, we used WGCNA analysis. Genes, which have similar trajectories using the WGCNAs clustering method, are grouped into modules. The modules were then correlated with three phenotypic endpoints, *i.e*., survival fraction of DA neurons; OCR, and locomotion ability at all time points (day 1, 3 and 6). We also added krill oil treatment as a parameter to be able to follow the directionality of correlation (Figure 5A). The correlation ranges from +1 (completely positively correlated) to -1 (perfect negative correlation), where a positive correlation indicates that increased gene expression results in an increase of the value of that parameter or vice versa.

Correlation values between all of the modules identified and defined parameters are shown as a heatmap (Figure 5A). Interestingly, different phenotypic endpoints were correlated with different sets of modules even though these phenotypes are, to some extent, functionally linked. The red module (comprising 1462 genes) has a strong positive correlation with the survival of the DA neurons (Figure 5A). The trajectory of the red module showed that the genes are downregulated in day 1 adults (Supplementary Figure 4). The module is functionally enriched for mitochondrial unfolded response and translation. The skyblue module, comprising 144 genes that are functionally enriched for the biological processes of lysosome and defense responses, was inversely correlated with DA survival, OCR and motility (Figure 5A) with a trajectory characterized by downregulation in day 3 animals (Supplementary Figure 4). For genes in the blue module, that strongly correlate with improved motility, the trajectory showed increased expression in day 6 animals. As some modules contained a number of genes too low for meaningful functional enrichment analyses, we grouped modules with similar trajectories (Supplementary Figure 4). Genes upregulated in day 1 animals (turquoise, white and saddlebrown) were enriched for basic cellular processes, such as DNA repair/replication, mRNA processing, and biogenesis. Interestingly, there was a strong functional enrichment for genes regulated by the transcription factors *cebp-1* (1.174e-22), *ceh-48* (5.53e-33), *efl-1* (1.649e-12), *ceh-28* (1.676e-11) (Figure 5B). Modules downregulated in day 1 animals (red, pink, dark grey) were enriched for biological processes related to ion transport across membranes, synaptic signaling, and transmission. Functional enrichment was found for mitochondrial unfolded response (UPR). Two transcription factors *elt-3* (9.006e-06) and *skn-1* (1.713e-02) were found as upstream regulators (Figure 5C). Enriched biological processes in modules with trajectories upregulated in day 3 animals (blue, tan, cyan, light cyan, violet, dark magenta, sienna) included neuron differentiation, cell communication, and cell projection organization. *tra-1* (1.822e-03) was identified as an upstream regulator of these gene sets (Figure 5D). Finally, trajectories upregulated in day 6 animals were dominated by biological processes related to immunity and defense, as well as protein metabolism (Figure 5E). Upstream regulators included *nhr-6* (4.79e-08) and *hlh-30* (3.71e-05). Thus, krill oil modulated separate gene expression programs in different stages of adulthood.

### Global gene expression changes induced by krill oil promote neuron survival

**Figure 6:**
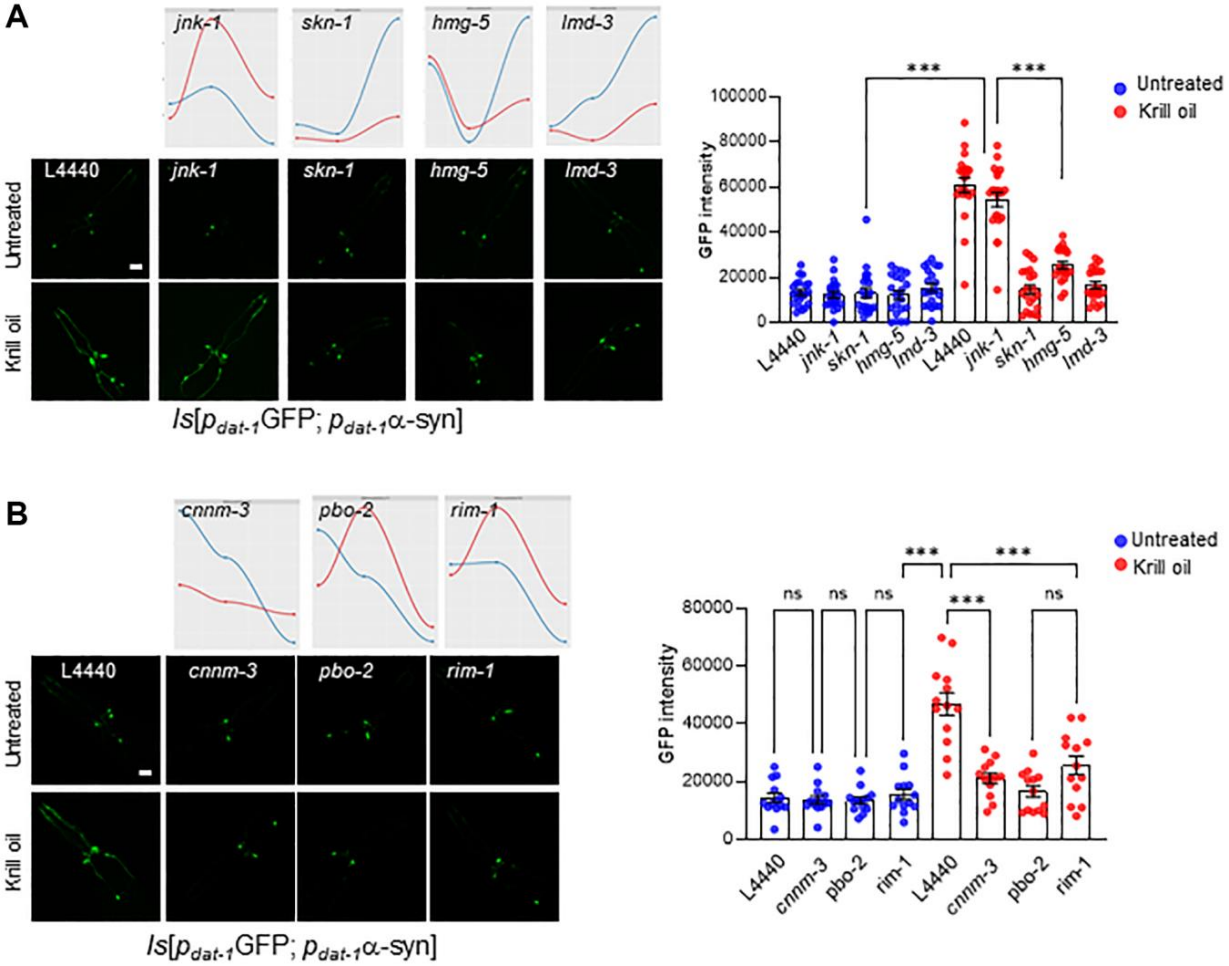
**Gene expression rewiring promotes dopaminergic neuron survival during aging.** (**A**) Representative images of the head region of PD animals at day 6 adulthood in control and krill oil treated animals following knockdown of *jnk-1*, *skn-1*, *hmg-5* and *lmd-3* by RNAi, Scale bar, 20 μm. Time series plot for same genes are show on top. The scatter dot plots represent GFP intensity of the CEPs dopaminergic neurons in day 6 PD nematodes following knockdown of *jnk-1*, *skn-1*, *hmg-5* and *lmd-3* (*n* = 20 individuals; Error bars, s.e.m; ^***^p < 0.001; one-way ANOVA followed by Bonferroni’s multiple comparison test). (**B**) representative images and scatter dot plots represents intensity of the dopaminergic neurons after depleting *cnnm-3*, *pbo-2* and *rim-1* in PD animals (*n* = 14 individuals, Scale bar, 20 μm Error bars, s.e.m; ^***^p < 0.001; one-way ANOVA followed by Bonferroni’s multiple comparison test), co-related with time series plot for same genes.

The gene expression signature in young adults appeared to reflect modulation of biological processes and transcription factors know to promote healthy aging. To test whether these changes in gene expression is indeed attenuating DA neuron degeneration in old animals, we scored the dopaminergic neuron survival after RNAi mediated knock-down (Figure 6A and 6B). Depletion of the central oxidative stress regulator SKN-1, which was downregulated in the krill oil treated animals (Figure 5C), the oxidative stress regulator LMD-3, and mitochondrial transcription factor HMG-5, led to abrogation of the neuroprotective effect of krill oil in the PD strain (Figure 6A). However, depletion of JNK-1, which was strongly upregulated in day 3 and 6 old animals, had no effect (Figure 6A). Upon depletion of *cnnm-3*, *pbo-2* and *rim-1* (human homologs of *CNNM3*, *PLCB4* and *RIMS1,* respectively), the neuroprotective effect of krill oil was abolished in the PD strain, while no effect was seen in the wild type background. Thus, this supports that krill oil induces changes in expression of several genes that directly promote DA neuron survival (Figure 6A and 6B).

### Krill oil improves dopamine dependent cognition in *C. elegans*

**Figure 7:**
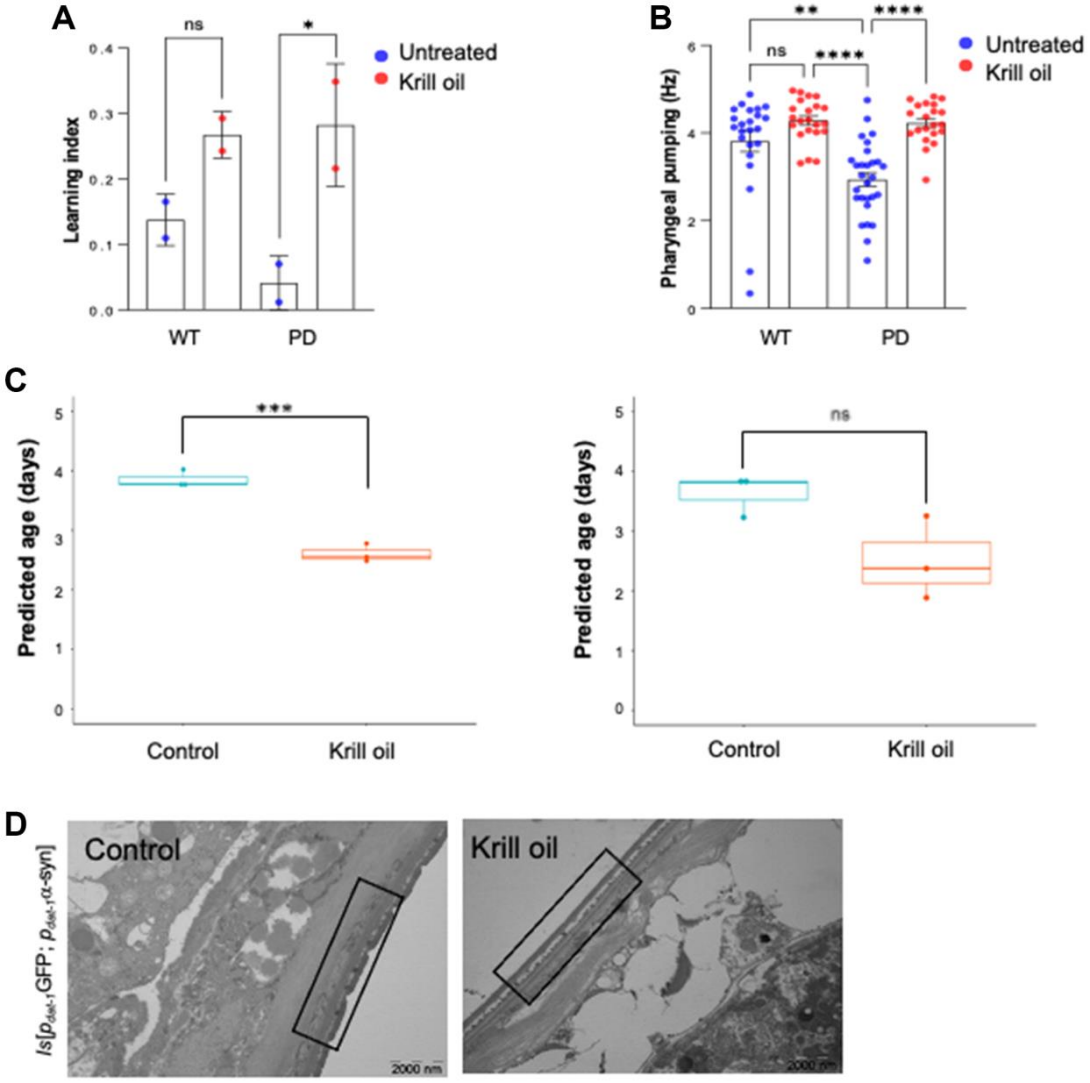
**Krill oil improves healthspan.** (**A**) The learning index was calculated from a positive association with butanone in wild type and PD animals at adult day 6 (*n* = 150 individuals, two independent experiments, error bars, s.e.m; NS, ^*^p < 0.05; one-way ANOVA followed by Bonferroni’s multiple comparison test). (**B**) Quantification of pharyngeal pumping frequency (Hz) monitored in wild type and PD animals using the NemaMetrix screenchip based assay. The column scatter plot represents pharyngeal pumping frequency of adult day 3 animals (*n* = 15–20 individuals, Error bars, s.e.m NS and ^**^P ≤ 0.001, ^****^p < 0.0001; one-way ANOVA followed by Bonferroni’s multiple comparison test). (**C**) Predicted biological age of wild type and PD animals in response to krill oil using BiTAge calculator. (**D**) TEM image of day 9 old in PD animals, boxed area represents the cuticle (scale bar 2000 nm).

To assess whether increased DA neuron survival and modulation of gene expression programs is accompanied by improved DA function, we measured dopamine dependent chemosensory cognition [41]. We found that krill oil-fed PD animals showed significant improvement of chemotaxis-associated learning capacity (Figure 7A). DA neurons regulate pharyngeal pumping [42], and the observation that krill oil fed PD animals displayed improved pharyngeal pumping, which is a commonly accepted healthspan metric in *C. elegans* (Figure 7B). Notably, wild type animals showed no significant benefit with respect to pharyngeal pumping nor cognition improvement (Figure 7A and 7B). Thus, krill oil improves dopamine dependent behaviors and cognition in *C. elegans*. To ask whether the neuroprotective effect might be relevant for other neurodegenerative diseases, we tested short-term associated memory in the *C. elegans* Alzheimer’s disease (AD) models [43]. A series of AD strains were used, including worms harboring neuronal Aβ pathology (CL2355), neuronal Tau pathology (BR5270 and CK12), as well as worms bearing both pathologies (UM0001) [44]. The chemotaxis-based memory-liked scores were improved in all AD worm strains fed with krill oil (Supplementary Figure 5A). The effect on senescence was assessed in a strain expressing Tau protein pan-neuronally (BR5270) [44], whereby animals fed with krill oil showed a 30 percent reduction in positive β-gal staining (Supplementary Figure 5B and 5C).

As aging is the main risk factor for PD, and many transcription factors known to affect lifespan in *C. elegans* emerged as regulators of the modules that associated with DA neuron survival, OCR, and motility, we were interested to test whether krill oil might modulate biological age estimates. Using the BiTAge calculator [33] krill oil appeared to significantly reduce the predicted biological age of day 6 old adults (Figure 7C). Finally, electron microscopy confirmed the younger appearance (Figure 7D), which is consistent with the improved healthspan.

## DISCUSSION

As aging has proved to be modifiable, there is a quest to identify anti-aging interventions [45]. Via genetic and pharmaceutical approaches, a variety of aging phenotypes can be significantly ameliorated, leading to extended lifespan and longer healthspan [43, 45, 46]. The possibility to reduce the burden of the major neurodegenerative diseases by counteracting aging, the most important risk factor of these diseases, gives a strong motivation to find novel healthspan interventions.

Krill oil has been shown to have neuroprotective properties in cells and rodent models [9, 12]. The underlying mechanisms have mainly been proposed to be via suppression of microglial activation, neuroinflammation, and oxidative stress [9]. However, studies evaluating how krill oil affects neuronal health through a natural life course is lacking. Here, we show that krill oil effectively protects DA neurons from age-related degeneration and reduces α-SYN aggregation in a *C. elegans* PD model. Consistently, krill oil improved dopamine dependent cognition and behavior, such as basal slowing response and pharyngeal pumping, in old PD animals.

Moreover, we uncovered that krill oil attenuates age-related neurodegeneration through rewiring of gene expression programs. Longitudinal analyses of global gene expression changes were used to identify gene sets that correlated with improvement of phenotypic read-outs, such as DA neuron survival. Functional enrichment of immunity and stress response pathways reflected a general anti-inflammatory effect of krill oil, exemplified with regulation of SKN-1, a master regulator of oxidative stress responses in *C. elegans* [47]. During conditions of oxidative stress, e.g., induced by mitochondrial dysfunction, SKN-1 is imported to nuclei, where it activates a coordinated transcription response [48, 49]. Interestingly, the gradual upregulation of *skn-1* expression with age was to a large extent abrogated in krill oil treated animals. Functional enrichment of SKN-1 regulated genes among the downregulated DEGs already in day 1 adults perhaps reflecting the potent antioxidant potential of astaxanthin. Similarly, LMD-3, an outer-mitochondrial membrane component involved in relaying the healthspan promoting effects of mild mitochondrial functions [20], had a similar trajectory as SKN-1. The role for SKN-1 in maintaining healthspan [48] and DA neuron survival upon oxidative stress and mild mitochondrial dysfunction [50, 51] are well described. Our data suggest that SKN-1 expression is tuned to a lower set-level in krill oil treated animals, perhaps reflecting a coordinated response to avoid pathogenic ROS signaling. Downregulation of SKN-1 - regulated genes were also consistent with functional data confirming that krill oil improves mitochondrial function. Consistently, genes regulated by krill oil were also functionally enriched for a wide array of metabolic processes. This is in line with a previous metabolomic analysis of *C. elegans* supplemented with krill oil, albeit from a different source than used in the present study [14]. Enrichment for biological processes involved in amino acid metabolism and protein metabolism in our study is consistent with increased levels of amino acids and derivatives (e.g., L-proline and L-phenylalanine) [14].

Interestingly, our data also suggest that krill oil might, in addition to the general oxidative stress related response, have more specific effects on synaptic transmission and neuronal functions. CEH-28, for example, is involved in regulation of synapse organization and neuron differentiation, in particular of cholinergic M4 motor neurons. It is possible that upregulation of CEH-28 responsive genes is related to choline in krill oil. RIM-1 is a positive regulator of cholinergic synaptic transmission in neuromuscular junctions [52]. PBO-2, phospholipase C beta expressed throughout the nervous system [53], facilitates synaptic transmission at neuromuscular junctions [53, 54] by stimulating acetylcholine release [55]. Upregulation of *rim-1* and *pbo-2* expression in day 3 and day 6 old animals is, thus, consistent with improved motoric function with advancing age. The *C. elegans* PD model further confirmed that protection of DA neurons depended on *pbo-2* and *rim-1*. In day 1 adults, genes regulated by the transcription factor CEBP-1 were upregulated. In *C. elegans*, CEBP-1 has been shown to activate a gene expression program that protects axons from degeneration [56]. Genes regulated by NHR-6, a transcription factor expressed in chemosensory neurons found to be important for functional plasticity [57], were enriched in day 6 adults. It is possible that this might be directly related to DA neuron survival, as loss of the mammalian ortholog (NR4A1) exacerbated DA loss in rats [58]. Expression of the NR4A subfamily was downregulated in PD patients [59] and it has been proposed that NR4A affect PD by modulating neuroinflammation [60]. Upregulation of Cyclin M protein 3, CNNM-3, was interesting in this regard, because it encodes a magnesium transporter expressed in hypodermis and rectum that has been shown to function in ROS homeostasis [61]. Its requirement for neuronal survival might suggest a systemic contribution to neuroprotection that might resemble the neuroinflammation response and microglial activation in mammalian cells.

Further functional relevance of these gene expression changes was shown by the striking suppression of age-related changes in cellular biomarkers of aging such as age-related loss of mitochondrial membrane potential and function by krill oil in both *C. elegans* and mammalian cells. Similarly, senescence and oxidative stress, as measured through 8-oxoG staining, was suppressed. Thus, it was interesting to see that several known regulators of lifespan appeared as upstream regulators in our RNA sequencing analyses. Genes downregulated in day 1 krill oil fed animals were enriched for ELT-3, which interacts with SKN-1 to activate a coordinated transcription response to oxidative stress [62] and thereby delays aging in *C. elegans* [63]. Similarly, EFL-1, promotes negative regulation of MPK-1 [64, 65]. Genes regulated by TRA-1, which regulates hermaphrodite lifespan and healthspan upstream of the classical lifespan regulating transcription factor DAF-16 [66], were enriched among the genes upregulated in day 3 animals. Together, this makes a strong argument that krill oil achieves neuroprotection by slowing down the aging process. Although not completely comparable formulations, krill oil was previously found to increase *C. elegans* lifespan [14], supporting that the reduction in biological age estimates found in the present study are functionally relevant.

## CONCLUSIONS

**Figure 8:**
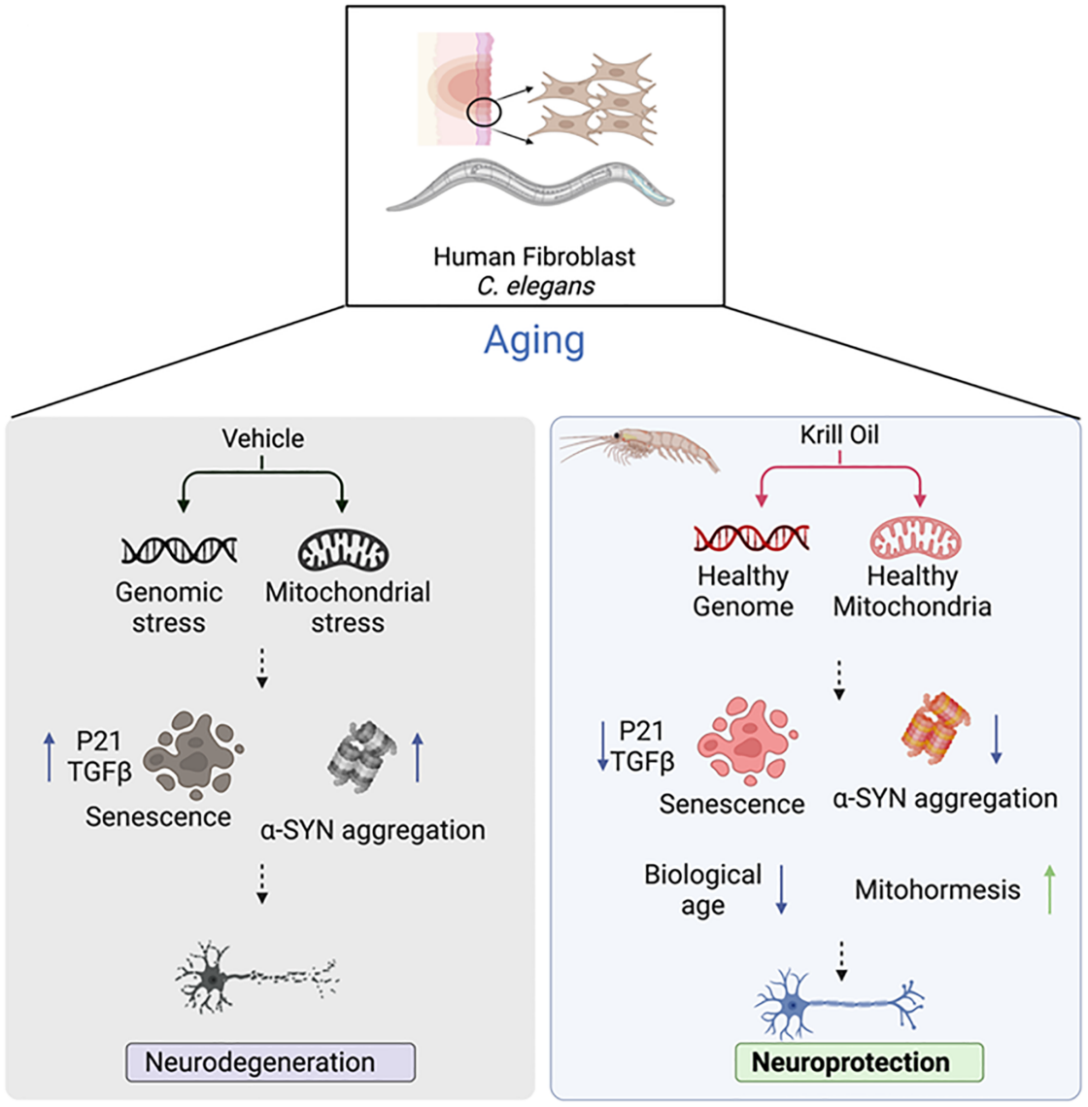
**Krill oil promotes neuron health by suppressing aging hallmarks.** The graphical representation demonstrates the benefit of krill oil and the pathways involved which leads to protection of DA neurons in *C. elegans* and human BJ fibroblast cells. The illustration was generated using https://biorender.com/.

We show that krill oil protects DA neurons from age-related degeneration and enhances dopamine-dependent behavior and cognition in *C.elegans* PD models (Figure 8). We show that Krill oil promotes healthy ageing by counteracting many processes that drive aging. Specifically, Krill oil suppresses accumulation of oxidative DNA damage, counteracts loss of mitochondrial membrane potential and function, suppresses senescence, and reduces α-SYN aggregation in old animals. Longitudinal RNA sequencing analyses showed that krill oil rewires global gene expression programs in mid-life and in old animals to attenuate several hallmarks of aging, results in remarkable protection of DA neuron survival in aging animals. Thus, krill oil supplementation might serve as a possible approach for healthy brain aging interventions.

## Supplementary Materials

Supplementary Figures

Supplementary Table 1
