# Supplementary material for: Krill oil protects dopaminergic neurons from age-related degeneration through temporal transcriptome rewiring and suppression of several hallmarks of aging
Source: Aging (Albany NY). 2022 Nov 9;14(21):8661–87. doi: 10.18632/aging.204375 (PMC9699765; doi:10.18632/aging.204375)
Supplement: Supplementary Figures [file aging-14-204375-s001.pdf]

SUPPLEMENTARY FIGURES

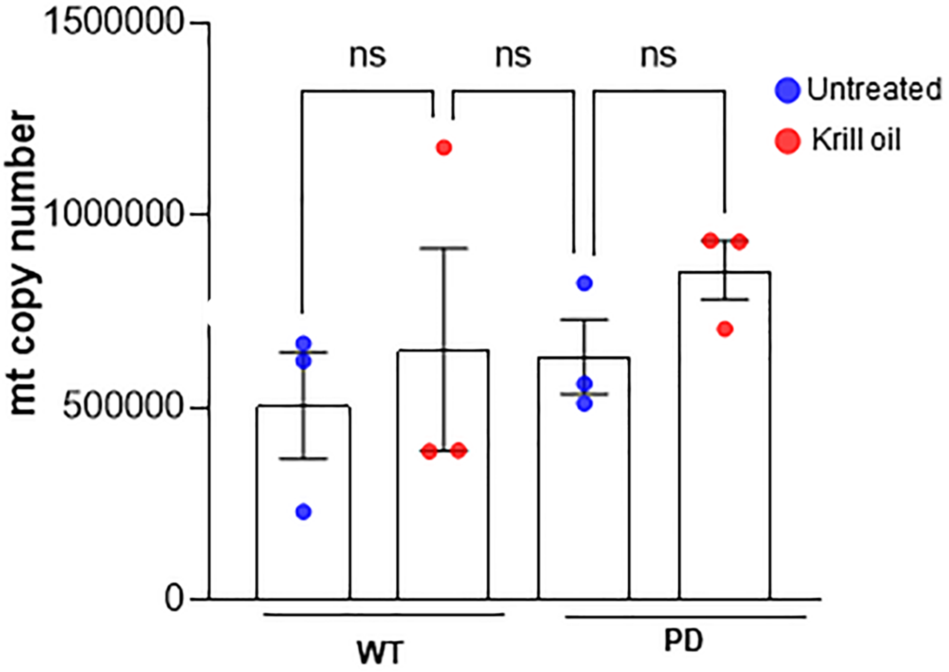

Supplementary Figure 1. Krill oil has no effect on mitochondrial copy number in *C. elegans*. mitochondrial copy number in day 6 old Wild type and PD animals in response to krill oil (n = 8 individuals, three independent experiments, Error bars, s.e.m; NS $p > 0.05$; one-way ANOVA followed by Bonferroni's multiple comparison test).

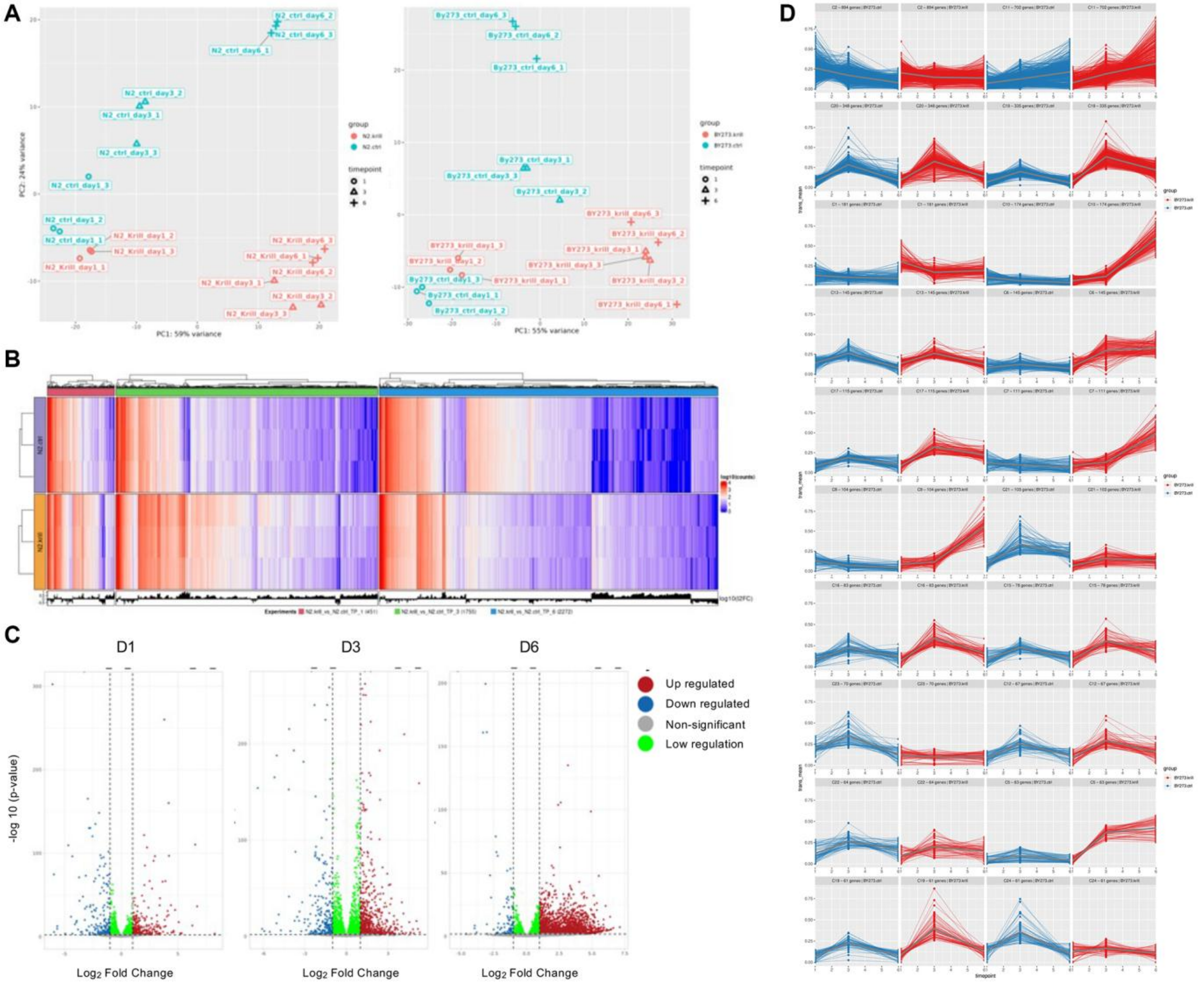

Supplementary Figure 2. Krill oil alters genes regulation in *C. elegans.* (**A**) Represents the PCA plot of wild type and PD animals in three separate time points day1, day3 and day6 animals. (**B**) Represents differentially expressed genes (DEG) in PD animals in presence and absence of krill oil treatment in day 1, day 3 and day 6 animal. (**C**) Represents the volcano plot of significant genes upregulated and downregulated in PD animals treated with krill oil in three different time point day1, day3 and day 6. (**D**) Represents clusters trajectories with upregulated and downregulated GOs in all clusters in PD animals treated with and without krill oil in three different time points day 1, day3 and day6.

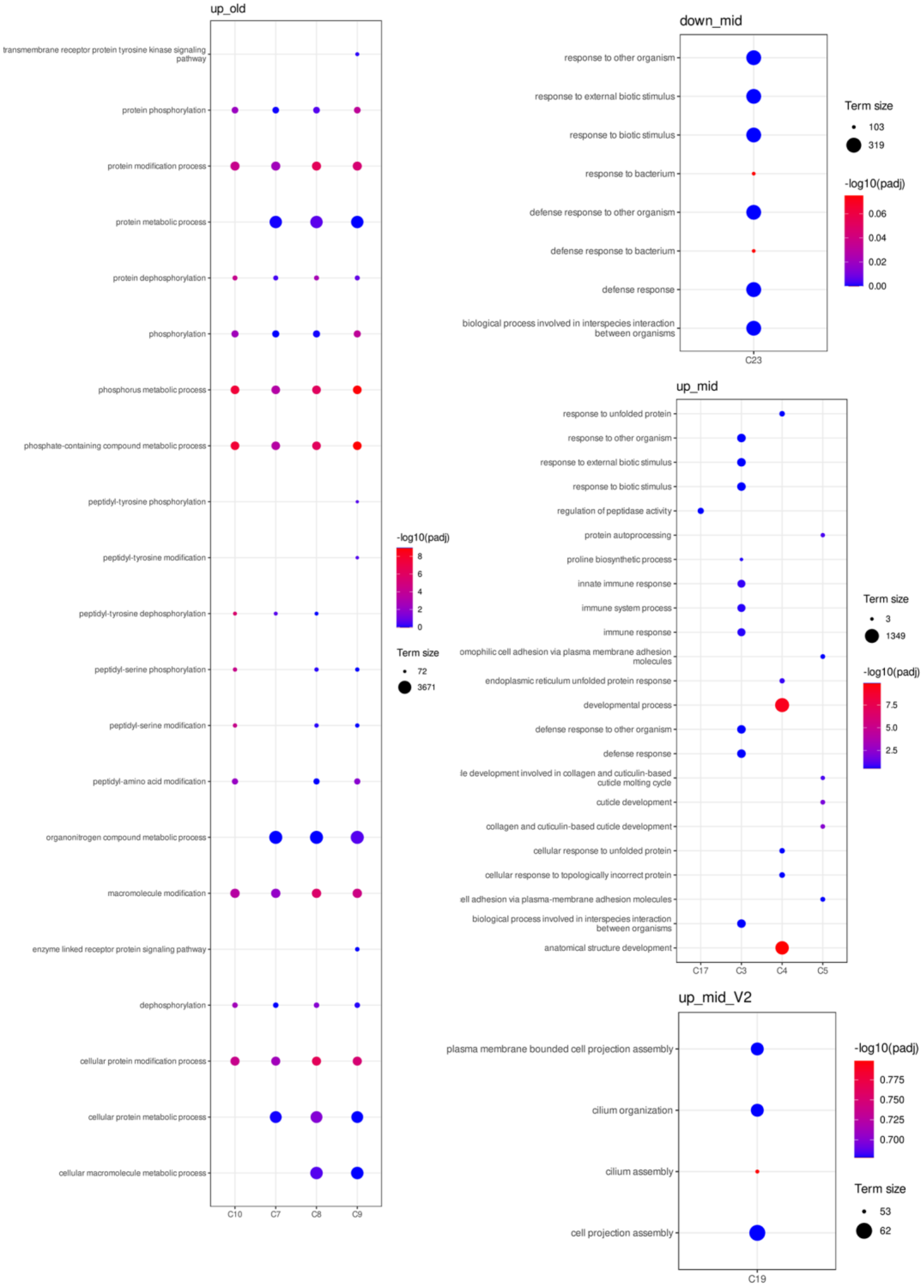

Supplementary Figure 3. Krill oil stimulates gene functions. Represents functional enrichment analysis performed on each cluster separately in PD animals treated with krill oil.

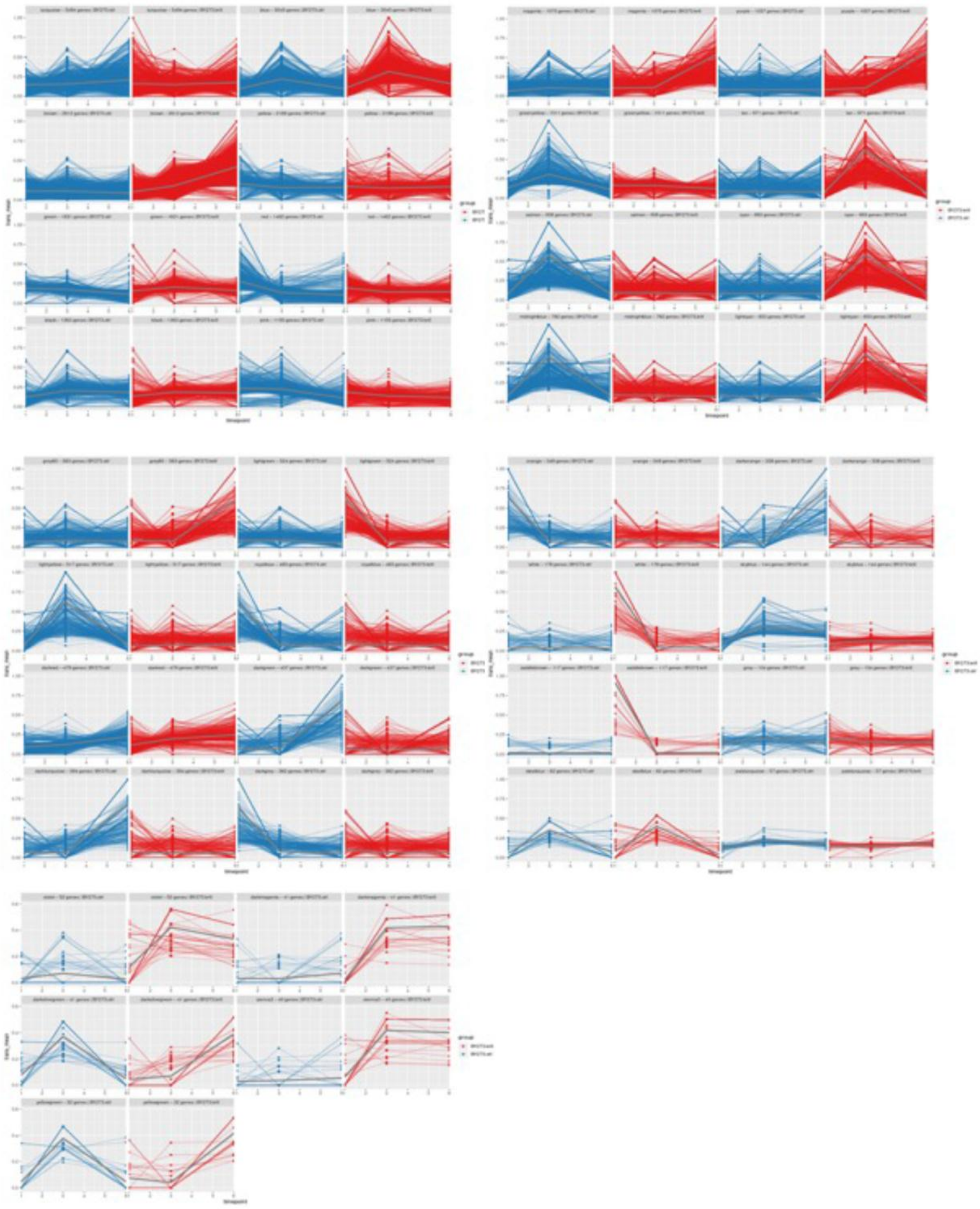

Supplementary Figure 4. Krill oil stimulates transcriptome. Represents the trajectories of all the modules generated by WGCNA analysis.

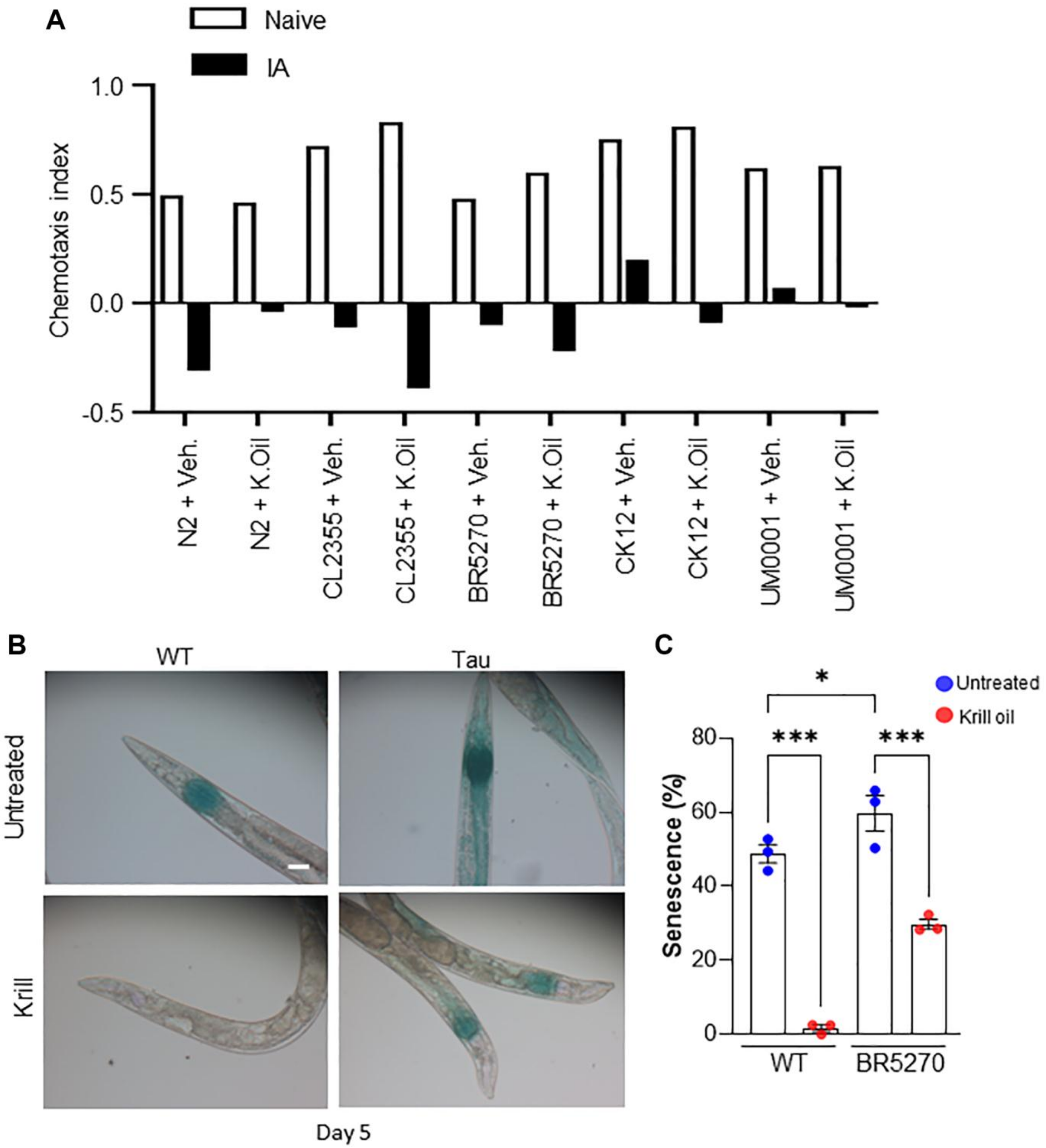

Supplementary Figure 5. Krill oil improves cognition and delays senescence in *C. elegans* AD model. (**A**) The learning index was calculated from a negative association with isoamyl alcohol (IA) in wild type and AD animals with and without Krill oil at adult day 1 (n = 150 individuals). (**B**, **C**) Representative images of the β-gal staining of the head region of wild type and AD animals at their adulthood of 5 days treated with and without Krill oil. The column scatter plot represents percentage of worms with positive senescence mark in three independent experiments (n = 50–100 individuals, column indicates mean, error bars, s.e.m, $^{***}p < 0.001$; one-way ANOVA followed by Bonferroni's multiple comparison test).
