# Supplementary material for: Krill oil protects dopaminergic neurons from age-related degeneration through temporal transcriptome rewiring and suppression of several hallmarks of aging
Source: Aging (Albany NY). 2022 Nov 9;14(21):8661–87. doi: 10.18632/aging.204375 (PMC9699765; doi:10.18632/aging.204375)
Supplement: Supplementary Table 1 [file aging-14-204375-s002.pdf]

SUPPLEMENTARY TABLE

Supplementary Table 1. List of PCR primer sequences used in mRNA expression analysis.

Gene	Species	Sequence (5′–3′)
p21	Human	Forward: GACACCACTGGAGGGTGACT<br>Reverse: CAGGTCCACATGGTCTTCCT
TGF-β	Human	Forward: TACCTGAACCCGTGTTGCTCTC<br>Reverse: GTTGCTGAGGTATCGCCAGGAA
